# Comparative Evaluation of Accuracy of Different Apex Locators: Propex IQ, Raypex 6, Root ZX, and Apex ID with CBCT and Periapical Radiograph—In Vitro Study

**DOI:** 10.1155/2021/5563426

**Published:** 2021-05-04

**Authors:** Okba Mahmoud, Mawada Hassan Awad Abdelmagied, Ahmad Hisham Dandashi, Bakr Nssaief Jasim, Hussam Alddin Tawfik Kayali, Saaid Al Shehadat

**Affiliations:** ^1^Clinical Sciences Department, Faculty of Dentistry, Ajman University, Ajman, UAE; ^2^Department of Preventive and Restorative Dentistry, College of Dental Medicine, University of Sharjah, Sharjah, UAE

## Abstract

**Objectives:**

This study aimed to validate the accuracy of working length (WL) measurements obtained with the newly introduced Propex IQ apex locator and to compare it with the latest generations of other electronic apex locators, CBCT, and conventional periapical radiographs by using the actual WL measurements obtained by using an endodontics microscope as a reference.

**Materials and Methods:**

Thirty-five extracted single-rooted human mandibular first premolars with curvatures from 10° to 20° were selected according to the inclusion and exclusion criteria and cut at the cementoenamel junction to achieve a standard reference point for WL determination. The actual WL was obtained by inserting a size-15 k-file in the root canal until the tip of the file was visible under an endodontic microscope. The definitive WL was measured using Propex IQ (Dentsply Sirona), Raypex 6 (VDW Dental), Root ZX (Morita), and Apex ID (Kerr Dental). In addition, radiographic WL was obtained using periapical radiographs and CBCT. One-way ANOVA was used for comparisons of the WL values, with a *p* value < 0.05. The percentage of success of each method for determination of the definitive WL was assessed using cross-tabulation and chi-square tests.

**Results:**

CBCT radiographs and Propex IQ apex locator yielded the most accurate WL measurements in comparison with the actual WL measurements (*p* < 0.05). Raypex 6, Root ZX, and Apex ID yielded more accurate WL measurements than conventional periapical radiographs (*p* < 0.05). Periapical radiographs yielded the least accurate WL measurements in comparison with the actual WL values (*p* < 0.05).

**Conclusions:**

Within the limitations of this study, the Propex IQ apex locator showed higher accuracy than Raypex 6, Root ZX, and Apex ID for WL determination in the root canal. Nevertheless, CBCT radiographs yielded the maximum accuracy for WL measurements.

## 1. Introduction

The goal of endodontic treatment is to eliminate infection and inflammation in the root canal and periapical area after irreversible pulp pathosis, and this is achieved by cleaning and shaping the canals to remove bacteria and debris and then filling the canal with three-dimensional root canal filling to prevent further infection in the apical area, alleviate pain, and preserve the tooth. Extrusion and the presence of core filling material beyond the root canal are potential irritants and they are considered as a possible cause of failure by some authors, whereas other authors consider them to be an indication of canal patency up to the apical foramen [1–3].

In clinical endodontics, the working length (WL) is defined as the distance between the reference point coronally and the physiologic foramen apically (ending at the apical constriction). Incorrect WL determination of the root canal can result in residual bacterial infection, which can lead to an enormous defect in the root end area, causing loss of the apical seal, endodontic treatment failure, and major flare-up problems [4].

Different methods have been used to locate the apical foramen and to measure the WL of root canals. These include conventional periapical radiographs, electronic apex locators, tactile evaluations, and other methods. The most common method of WL measurement is based on periapical radiographs alone, wherein the clinician uses these radiographs to visualize the extent of a file inserted in the canal and its relationship to the radiographic apex. However, this procedure is associated with multiple limitations, including subjectivity, image magnification, distortion errors, exposure of the patient to radiation, and superposition of anatomical structures [4]. The practice of estimating the WL by measuring the length of the root from the radiographic apex to the crown and then reducing 0.5–1 mm from the measurement has also been reported to be unreliable and inaccurate due to distortion of radiographic images [5–7].

Cone-beam computed tomography (CBCT) is an important technique that was introduced to dentistry in 1998 and has shown high potential for clinical applications with greater accuracy than periapical radiography [8]. CBCT has been shown to contribute to treatment planning, diagnosis, treatment, and prognosis of different diseases, in addition to its importance in research [9, 10]. CBCT images can show the root canal angles, height of the curvature, and location of the major foramen, which are not identifiable with sufficient precision in periapical radiography [11, 12].

The development and production of electronic apex locators for locating the canal terminus are a major innovation in root canal treatment. An electronic root length measurement method was first suggested by Custer [13] in 1918, after which the idea was revisited by Suzuki in 1942 [14]. However, it was Sunada [15] who, in 1962, used these principles to build a simple device that relied on direct current to detect the WL. Subsequently, electronic apex locators have undergone substantial improvements that have greatly increased their accuracy and adaptability.

Sunada stated that the apical constriction is the most important anatomical landmark because it has a resistance of 6500 Ω, which confers it with unique electronic characteristics [15]. Apex locators generate a direct current of known voltage (V) and include an ammeter that measures the intensity (I) of the current after it passes through the file and is recaptured by the labial hook [15]. An electronic component calculates the V\I ratio and deducts the resistance at the level of the canal where the instrument is located. The screen displays 0 when the resistance is 6500 Ω, which is how the clinician estimates that the tip of the file is at the apical constriction [15].

Although apex locators function with the same principle, the areas detected by different devices may differ. Whereas most manufacturers' manuals state that the devices detect the apical constriction, Morita (Dentaport ZX) suggests that their device detects the apical foramen and not the constriction. They also advised that the operator should stop advancing the file when the reading shows 0.5 on the screen in order to locate the constriction [16].

Apex locators showed equal or higher accuracy than radiographic in many *in vivo*, *ex vivo*, and *in vitro* studies [17–19]. These locators are useful when the apical portion of the canal system is hidden by some anatomical structures. Moreover, they help reduce the treatment time and radiation dose, which may be higher with conventional radiographic methods. However, the main problems associated with the use of electronic apex locators are that they cannot be used in cases of perforations, patients with cardiac pacemakers, and fractures of the root and that their accuracy is questionable in cases of root resorption, immature apices, swelling, and hemorrhage [20, 21].

The current study aimed to examine the accuracy of Propex IQ, a recently introduced electronic apex locator for which no accuracy data from *in vitro* or *in vivo* studies are currently available in the literature, and to compare it to the latest generation of other commercially available apex locators. Furthermore, the accuracy of these apex locators was compared to those of other commonly used methods for determining WL, namely, periapical radiography and CBCT.

## 2. Materials and Methods

The sample size (*n*) was calculated using an online Statistics Calculator link, and an a priori sample size calculator for Student's *t*-test was used to estimate the minimum sample size for the one-tailed *t*-test study, considering a probability level of 0.05, anticipated effect size of 0.9 based on similar studies, and a statistical power level of 0.8. The representative sample size was 35 teeth.

Thirty-five extracted human mandibular first premolars with curved and single root canals were kept in 5.2% sodium hypochlorite for 2 h and then stored in hydrogen peroxide solution until use in this study. Each tooth was marked at the cementoenamel junction (CEJ), placed inside a special acrylic mold, and stabilized by wax. Then, the crown of each tooth was cut at the CEJ by using a saw machine (IsoMet 1000 Precision Cutter; Buehler, Düsseldorf, Germany) to provide a standard reference point for all WL measurements.

**Figure 1:**
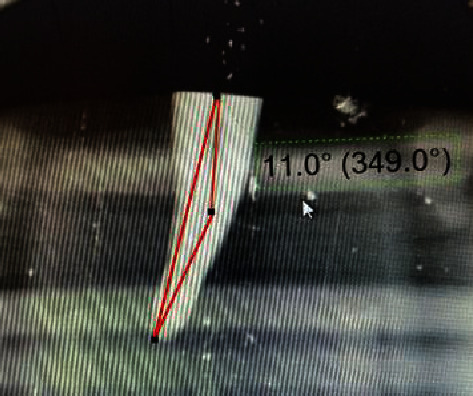
Lower first premolar with a 11° curvature that matched the teeth selection criteria in this study.

Periapical radiographs were taken for all teeth preoperatively to evaluate the curvature (10°–20°) and to check for any internal defects (Figure 1). Patency was checked with a size-10 k-file (Dentsply Maillefer, Ballaigues, Switzerland). The selected teeth were cleaned using an ultrasonic dental scaler (Guilin Woodpecker Medical Instrument Co., Ltd., China) to remove any debris from the root surface. Teeth were also examined under an endodontic microscope at 20x magnification (Extaro 300; Zeiss, Germany) to determine the apex maturity and root surfaces and to detect possible fractures or any defects as part of the inclusion and exclusion criteria (Table 1).

**Figure 2:**
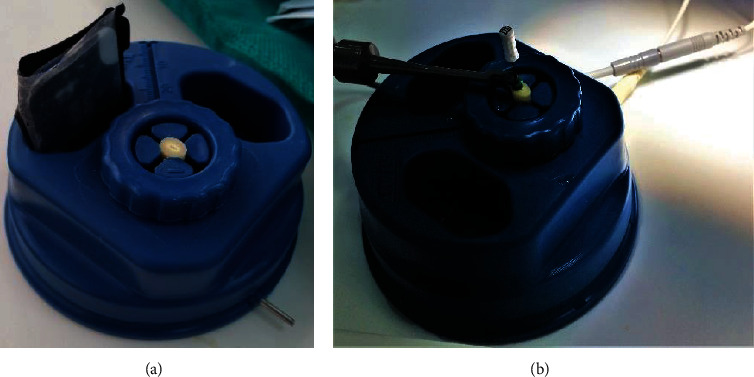
Protrain mold designed to simulate the oral environment of the extracted teeth.

Root canals were irrigated with 5 mL of 5% sodium hypochlorite (NaOCl, Werax, Izmir, Turkey). Before starting the WL measurements, each tooth was placed inside the Protrain mold (Simit, Italy), which is a special mold designed to simulate the oral environment for extracted teeth. This mold facilitates standardization by allowing a standard tooth position, standard X-ray imaging for all teeth, a standard SLOB technique, and a standard pathway for apex locators to complete the electrical circuit (Figure 2).

### 2.1. Actual WL Determination Using a Microscope

The actual WL was measured as a control value by inserting a size-15 K-file (Dentsply Maillefer, Ballaigues, Switzerland) with a double stopper to decrease the chance of stopper movement during measurements. The file was inserted in the root canal until its tip could be observed at the apical foramen under a microscope and then withdrawn 0.5 mm, after which the length between the file tip and reference point was measured with a digital caliper (Allendale Electronics Ltd.). Each measurement was repeated three times by three independent authors, and the mean value was recorded as the representative measurement of that sample.

### 2.2. Radiographic WL Determination Using Periapical Radiographs

After placing the tooth in the Protrain mold, two conventional periapical radiographs were taken for each tooth. The first radiograph was used to evaluate the tooth on the basis of the tooth selection criteria and to determine the radiographic tooth length and the estimated WL (whole tooth length—0.5 mm). The second radiograph was also taken using the Protrain mold after inserting a size-15 K-file up to the estimated WL to obtain the radiographic WL, which was calculated as the total file length inside the canal + the distance between the tip of the file on the radiograph and the root end (determined using an internal digital ruler of the digital radiograph software)—0.5 mm.

### 2.3. Electronic WL Determination Using Apex Locators

Four well-known electronic apex locators were used in this study: Propex IQ (Dentsply Maillefer, Ballagiue, Switzerland), Raypex 6 (VDW, Munich, Germany), Root ZX (J Morita Corp., Kyoto, Japan), and Apex ID (Sybron Endo). Selected and prepared teeth were placed inside the Protrain mold; the roots were embedded in the mold, leaving approximately 5 mm of the coronal root surface exposed; and the labial clip of the apex locator was attached to the mold (Figure 2).

To obtain the electronic WL measurement, a size-15 K-file with double stoppers was connected to each apex locator and used to determine the electronic WL in each root canal. The canals were irrigated with 5.0% NaOCl. Subsequently, cotton pellets and paper points were used to dry the tooth surface and to eliminate the excess irrigation solution, after which Propex IQ, Raypex 6, Root ZX, and Apex ID were used. Each file was attached to the apex locator file holder and was gradually introduced inside the canal while carefully monitoring the apex locator screen. The file was progressed in each canal until the apex locator screen indicated that the file was outside the root canal (beyond the WL), which was accompanied by a warning sound and red bars. The file was then regressed very slowly to the point where it showed the apical constriction and indicated the WL where the endodontic treatment should terminate. Each electronic apex locator was used according to the manufacturer's instructions. Three measurements were obtained by three different authors, and the mean of these three consecutive measurements was recorded as the representative electronic WL measurement of each canal for the corresponding device.

### 2.4. Radiographic WL Determination by CBCT

Two cone-beam computed tomography (CBCT) images were acquired for each tooth (Planmeca Promax 3D, Finland). Each group of six teeth was inserted separately in a special mold made from putty to facilitate the imaging procedure in the CBCT device. The first image was used to determine the CBCT radiographic tooth length and the estimated WL (whole tooth length—0.5 mm). The second image was obtained after inserting a size-15 K-file to the exact estimated WL of each tooth to obtain the CBCT radiographic WL: total file length inside the canal + the distance between the tip of the file and the root end (measured by the internal digital ruler of CBCT software)—0.5 mm.

### 2.5. Statistical Analysis

The collected data were analyzed using Statistical Package for Social Sciences (SPSS) for Windows software, version 20 (SPSS Inc., Chicago, IL, USA). One-way analysis of variance (ANOVA) was used in this study with *p*values < 0.05. The percentage of success of each electronic apex locator in finding the exact WL was assessed using cross-tabulation and chi-square tests.

## 3. Results

**Figure 3:**
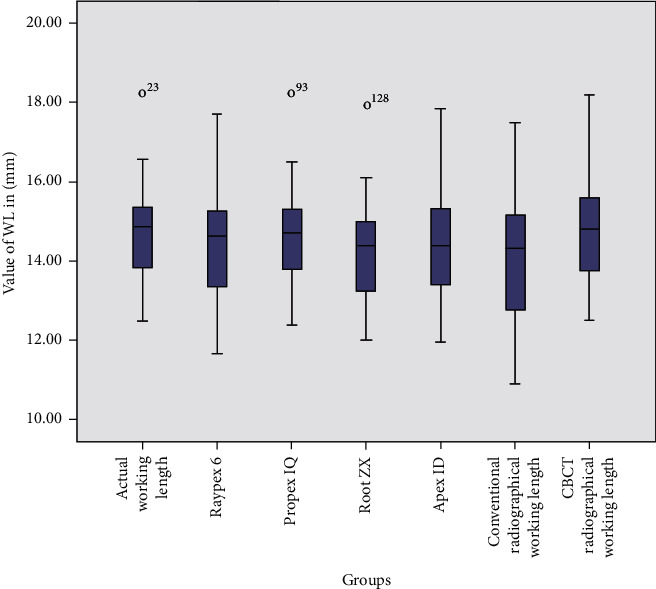
Boxplot showing the mean WL values in mm for four different electronic apex locaters and conventional periapical radiographs and CBCT scans in comparison with the actual WL values.

The mean value of the actual WL measurements obtained with the endodontic microscope was 14.74 ± 1.23 mm. CBCT yielded WL measurements closest to the actual WL (mean = 14.70 mm), followed by Propex IQ (mean = 14.66 mm). The least accurate WL measurement was obtained using conventional periapical radiographs (mean = 14.01 mm) (Figure 3 and Table 2).

The WL values obtained with the four electronic apex locators were not significantly different; however, the WL measurements obtained using conventional radiographs were significantly different from the actual WL values (*p* < 0.010) (Table 3).

The results of this study were divided into three groups to validate the WL values obtained by each electronic apex locator by comparing the differences between each device and the actual WL values separately (Table 4). Positive values indicated measurements that were overextended from the actual WL, while negative values indicated measurements that were underextended from the actual WL, whereas values within 0.5 mm from the actual WL were considered coinciding measurements.

The WL measurements obtained using CBCT radiographs and Propex IQ apex locator showed all WLs within ±0.5 mm from the actual WL, while Raypex 6, Root ZX, and Apex ID showed most WLs within ±0.5 mm. However, some WLs obtained with these locators were <0.5 mm and >0.5 mm from the actual WL, except for Root ZX, which had no WL in the <0.5 mm category. Lastly, most radiographic WLs were >0.5 mm from the actual WL.

## 4. Discussion

The results of this study highlight the differences between various methods of WL determination, in addition to providing comparative data for the different commercially available electronic devices for measuring the WL in curved single-rooted canals. Correct and definitive determination of the WL is the primary factor for successful endodontic treatment. The histological results after root canal treatment have been shown to be superior when instrumentation and obturation are limited to the apical foramen than beyond this anatomical landmark. Thus, accurate determination of the location of the intended apical constriction is an important factor in the success of root canal treatment [22].

In this study, we used four different well-known apex locators and both conventional periapical radiographs and CBCT to compare the WLs measured using these techniques to the actual WL, which was determined by a microscope for each tooth separately. No published literature has investigated the use of the Propex IQ electronic apex locator in determining the WL, since it was recently introduced in the market. Therefore, we examined the accuracy of these devices in curved single-rooted extracted mandibular first premolars [23]. The results of the current study demonstrated that WL measurements using CBCT radiographs and the Propex IQ apex locator were the most accurate, while conventional radiographs yielded the least accurate WL measurements. The WL measurements obtained with Raypex 6, Root ZX, and Apex ID showed acceptable accuracy in comparison with those obtained with Propex IQ and better accuracy than measurements obtained with conventional radiographs. This finding was in agreement with the study conducted by Adriano et al., who performed in vitro comparisons between apex locators and direct and radiographic techniques for determining the root canal length in primary teeth [17]. On the other hand, the findings of our study contradict those reported by Midhun Mohan and Susila Anand, who found that electronic apex locators are not superior to conventional radiographs in determining WL [24].

Janner et al. published the first study that compared the accuracy of WL measurements using preexisting CBCT scans with those obtained using standard techniques such as electronic apex locators, and they observed a high correlation between both methods [25]. Tchorz et al. found that CBCT is a useful tool for planning endodontic treatment, visualizing complex root canal anatomies, and estimating root canal length [26]. However, the application of CBCT exclusively for root canal length measurement is not yet recommended, since the benefits may not always outweigh the potential risks of the additional radiation [27]. In this regard, each endodontic patient should be evaluated individually, and when more evidence is needed, CBCT should only be considered when normal imaging does not yield adequate information for proper management of the case [28].

Our data also demonstrated that CBCT allowed better WL determination than electronic apex locators, which contradicts the findings reported by González‐Rodríguez et al., who showed that electronic measurements were more reliable than CBCT scans for WL determination [18]. This difference may have occurred because identification of the apical constriction required higher magnification, and a stereomicroscope (920–25) was used in their study [18]. Jorge Paredes Vieyra et al. also reported that electronic apex locators showed higher accuracy and predictability than digital radiographs, and there were no significant differences in accuracy between Root ZX, Raypex 6, and Apex ID [19], whereas in another in vitro study, Root ZX exhibited higher accuracy than Apex ID in determining the WL of curved molar canals [29].

Yolagiden et al. conducted a study to compare four electronic apex locators in detecting a position 0.5 mm short of the major foramen, and their results showed that Apex ID allowed acceptable determination of the WL and its accuracy was similar to those of Raypex 5 and Raypex 6 [30]. Nevertheless, a −0.5 mm difference in the accuracy of electronic apex locators has been considered acceptable in various studies [29, 31], while others considered an acceptable range of ±1.0 mm [32].

In summary, the accuracy of WL measurements and the comparisons among electronic apex locators, radiographs, and CBCT images remain topics of debate. Since the existing data are insufficient, more research with a larger variety of methods and techniques is required to emphasize the improvements achieved with these devices for better endodontic practice. However, conventional periapical radiographs appear to have lower accuracy than all the electronic apex locators evaluated in this study.

## 5. Conclusions

Within the limitations of this study, CBCT-based radiographic measurements were the most accurate method for determining the WL of the root canal. However, the WL measurements obtained by Propex IQ were more accurate than those obtained with the other electronic apex locators and very close to those obtained with the CBCT radiographs. Conventional radiographs were less accurate and cannot be used to determine the WL of the canal. Although Raypex 6, Root ZX, and Apex ID showed no significant differences in their accuracies for determination of the WL of root canals, they were not as accurate as Propex IQ.

## Figures and Tables

**Table 1:** Inclusion and exclusion criteria used in this study.

Inclusion	Exclusion
Lower first premolars	Teeth other than lower first premolars
Curved canal with a curvature between 10 and 20°	Curved canal of less than 10° or more than 20°
Sound, noncracked, nonworn, or nonfractured tooth	Worn, carious, resorbed, cracked, fractured, filled, and malformed teeth
Initial apical file must be K-file size 10 or 15	Initial apical file more than K-file size 15
No calcifications or internal defects of the root canal	Calcified canal or pulp stones
Single-rooted teeth	Multirooted teeth

**Table 2:** Mean WL values in mm obtained for each group in comparison with the actual WL (control group) values.

Groups	Mean (mm)	SD (mm)	Minimum (mm)	Maximum (mm)
Actual WL *(control group)*	14.73	1.22	12.50	18.24
Raypex 6	14.39	1.24	11.65	17.70
Propex IQ	14.65	1.23	12.37	18.20
Root ZX	14.35	1.23	12.00	17.95
Apex ID	14.42	1.26	11.94	17.85
Conventional radiographs	14.01	1.57	10.90	17.48
CBCT radiographs	14.70	1.23	12.50	18.20

**Table 3:** One-way ANOVA for comparison of the WL values measured obtained using the different techniques. df = 9, F value = 1.53. The actual WL value was closest to the value obtained with CBCT (*p* < 0.90), followed by Propex IQ (*p* < 0.80). However, the actual WL value was significantly different from that obtained with conventional radiographs (*p* < 0.010).

Multiple comparisons (LSD) (post hoc test)		*p* value
Actual WL	Raypex 6	0.28
	Propex IQ	0.80
	Root ZX	0.23
	Apex ID	0.31
	Conventional radiographs	0.01
	CBCT radiographs	0.90

**Table 4:** Number of teeth (*n*) and frequencies of WL measurements (%) that were less, more, or within 0.5 mm from the actual WL. Both groups (<0.5 mm and >0.5 mm) were considered to indicate errors in determining the WL of each tooth. WL measurements obtained using CBCT radiographs and Propex IQ showed no errors, whereas the other WL determination methods showed several errors.

WL determination method	(<0.5 mm) *n* (%)	(±0.5 mm) *n* (%)	(>0.5 mm) *n* (%)
Propex IQ	Nil	35 (100%)	Nil
CBCT radiographs	Nil	35 (100%)	Nil
Raypex 6	1 (2.86%)	25 (71.43%)	9 (25.71%)
Root ZX	Nil	25 (71.43%)	10 (28.57%)
Apex ID	1 (2.86%)	24 (68.57%)	10 (28.57%)
Conventional radiographs	2 (5.71%)	9 (25.71%)	24 (68.57%)

## Data Availability

The data used to support the findings of this study are available from the corresponding author upon request.
